# The antiapoptotic effects of conditioned medium from bone marrow-derived mesenchymal stromal stem cells on cyclophosphamide-induced testicular damage in rat: An experimental study

**DOI:** 10.18502/ijrm.v22i2.15706

**Published:** 2024-03-25

**Authors:** Zeynab Esmailpour, Soheila Madadi, Maryam Baazm

**Affiliations:** ^1^Students Research Committee, Arak University of Medical Sciences, Arak, Iran.; ^2^Department of Anatomy, School of Medicine, Alborz University of Medical Sciences, Karaj, Iran.; ^3^Department of Anatomy, School of Medicine, Arak University of Medical Sciences, Arak, Iran.; ^4^Molecular and Medicine Research Center, School of Medicine, Arak University of Medical Sciences, Arak, Iran.

**Keywords:** Bone marrow mesenchymal stem cells, Cyclophosphamide, Conditioned medium, Apoptosis, Spermatogenesis.

## Abstract

**Background:**

Cyclophosphamide (CP) has some negative effects on the reproductive system. Stem cells and their metabolites are being utilized to enhance fertility after chemotherapy.

**Objective:**

This study aimed to investigate the impact of conditioned medium (CM) derived from bone marrow mesenchymal stromal stem cells (BM-MSCs) on the toxic effects of CP on testicles.

**Materials and Methods:**

BM-MSCs were isolated, a CM was collected and 25-fold concentrated. 24 male Wistar rats (8 wk, 200–250 gr) were randomly divided into following groups: control, CP, CP+ Dulbecco’s Modified Eagle Medium (DMEM), CP+CM. CP was given at a single dose of 100 mg/kg. 2 wk after the CP administration, CM was injected into the testicular efferent duct. Sperm parameters, testicular histopathology, and the level of testosterone were analyzed 2 months after treatment. The expression of B-cell lymphoma 2 (*Bcl2*) and *Bcl2*-associated X protein (*Bax*) genes were evaluated by real-time polymerase chain reaction.

**Results:**

CP had a negative effect on testis histology (p < 0.001) and sperm quality (p < 0.001). It changed the expression of genes associated with apoptosis (p < 0.001). Treatment with CM reduced the expression of *Bax* (p < 0.001), while significantly increasing the expression of *Bcl2* (p = 0.01). It improved sperm count (p = 0.03), viability (p < 0.001), motility (p < 0.001), spermatogonial count (p < 0.001), and epithelial thickness of testicular tubules (p = 0.02).

**Conclusion:**

These findings suggest that CM produced from BM-MSCs may be valuable for therapeutic approaches in reproductive medicine and may lessen the side effects of CP.

## 1. Introduction

Antineoplastic agents such as cyclophosphamide (CP) are frequently employed to treat a variety of malignancies and autoimmune diseases. However, their broad clinical application is limited due to undesirable side effects, such as toxicity to humans and experimental animal reproduction (1). CP exerts its cytotoxic effects through the production of 2 stable metabolites: phosphoramide and acrolein (2). It generates reactive oxygen species, which can promote apoptosis and cause damage to testicular tissue. Oligozoospermia, azoospermia, and reduced gonadotropin production are the other side effects of CP, which eventually leads to infertility (1).

Stem cell transplantation, such as spermatogonial stem cells or mesenchymal stem cells (MSCs), is recommended to preserve testicular function after cancer treatment. Bone marrow mesenchymal stromal stem cells (BM-MSCs) are commonly used in regenerative medicine. However, cell therapy has certain issues, including ethical concerns, contamination with cancer cells, and the risk of infectious transmission (3). BM-MSCs can secrete various substances that can modify the microenvironment (4). Their capacity to release numerous factors crucial for tissue regeneration is mainly responsible for their therapeutic effectiveness (5).

This complex of bioactive chemicals comprises various growth factors, cytokines, and chemokines collectively known as secretome, and is described as a conditioned medium (CM) (6). The CM possesses various biological properties, such as anti-inflammatory, antioxidant, and anti-apoptotic capabilities, and can be obtained during in vitro BM-MSCs culture (7). Therefore, BM-MSCs CM, which contains the secreted products of BM-MSC, can serve as an alternative approach to cell therapy (8). CM derived from BM-MSCs has been shown to minimize spermatogonial stem cell apoptosis, increase intercellular adhesion molecule expression (9), and restore spermatogenesis in mice treated with busulfan (10). Moreover, because CM contains growth factors and antioxidants, adding it to the sperm freezing medium has been shown to increase the indicators linked to the quality of the frozen-thawed sperm cells (11). Testicular cell-CM can also be a promising approach for in vitro differentiation of embryonic stem cells into male germ cells (12, 13). Injecting BM-MSCs or their CM into the ovaries of rats that had received CP has been shown to have a similar effect in enhancing ovarian structure and function (14).

CM contains some cytokines with anti-apoptotic properties, such as interleukin-6, interleukin-10, and tumor necrosis factor-alpha. These cytokines can activate intracellular signaling pathways that inhibit apoptotic signaling and promote cell survival (15). As mentioned, CP induces oxidative stress by producing active oxygen species such as superoxide anions and hydroxy radicals, which reduce the activity of antioxidant enzymes such as superoxide dismutase and catalase, and promotes apoptosis (16).

In this study, we hypothesized that substances produced by BM-MSCs could potentially alleviate testicular damage, reduce apoptosis, and modulate gene expression related to apoptosis, such as *Bax* (proapoptotic) and *Bcl2* (antiapoptotic), following chemotherapy. Therefore, this study aimed to investigate the effectiveness of CM released by BM-MSCs in mitigating the detrimental effects of CP on testicular function, structure, and apoptosis gene expression.

## 2. Materials and Methods

The National Research Council's guidelines were followed during this research. Animals were kept in climate-controlled environments with a 12-hr light/dark cycle and free access to food and drink.

### Experimental design

In this experimental study, 24 adult male Wistar rats (8 wk, 200–250 gr, Pasteur, Iran) were randomly divided into 4 groups (n = 6): 1: control, 2: CP (100 mg/kg) (17), 3: CP + Dulbecco's Modified Eagle Medium (DMEM; 100 μl) (18), and 4: CP + CM of BM-MSCs (100 μl).

### Isolation and culture of bone marrow mesenchymal stem cells and preparation of CM

BM-MSCs that are proven to have remarkable contributions to reparative were isolated from 8-wk male Wistar rats, as previously reported (19). Concisely, bone marrow was isolated by flushing the femur and tibia with DMEM (Gibco, Germany) that contained 10% fetal bovine serum (FBS; Gibco, Germany) and 1% penicillin-streptomycin (Gibco, Germany) under sterile conditions and centrifuged at 1800 rpm for 10 min. Then, the cell pellets were resuspended and cultured in 25-cm² culture flasks and incubated at 37 C in a humidified chamber filled with 95% air and 5% CO ${\;}_{2}$ . After 48 hr, the non-adherent cell-containing medium was removed from the flask by washing with phosphate buffer saline (PBS; Sigma, Germany), and replaced with a fresh medium. The medium was changed every 3 days. When the cells were 80% confluent, they were detached using 0.25% trypsin/ethylenediaminetetraacetic acid (Gibco, Germany) and continued to be cultured up to the fifth passage. In passage 3, to confirm the capacity of cultured BM-MSCs, they were differentiated into bone, cartilage, and adipose tissue (data not shown), and the surface markers for stem cells were analyzed by flow cytometry. BM-MSCs that had reached a confluence level of more than 80% were re-fed with serum-free and antibiotic-free DMEM. In this setting, 1 × 10^6^ BM-MSCs were cultured for 48 hr. Then, the CM was collected from the cultured cells and concentrated 25-fold using ultrafiltration units with a 10 kDa molecular weight cutoff. For later usage, the concentrated medium was kept at -80 C.

### Flow cytometry

After trypsinizing BM-MSCs in passage 3, the 10⁶ cells were fixed for 30 min in a neutralized 2% paraformaldehyde solution. Then, 2 PBS washes were performed on the fixed cells. The following antibodies were used to incubate the fixed cells for 20 min at 4 C in the dark: CD34 and CD45, the hematopoietic surface markers, as negative controls, as well as CD44 and CD90, the BMSCs surface markers, (all from Abcam, USA) as positive controls. The specific fluorescence of 1 × 10 ⁴ cells per sample was acquired and analyzed by flow cytometric software (19).

### Treatment 

2 wk after theCP injection, the animals were anesthetized using an intraperitoneal dose of 100 mg/kg ketamine and 10 mg/kg xylazine (both from Alfasan, Netherlands). Then, BM-MSC-CM or DMEM was injected into the left testes through the efferent duct of CP-treated rats. To demonstrate the medium injection process, trypan blue was employed. The amount of DMEM or CM injected into the efferent duct of each rat was according to the amount used in the stem cell transplantation to the rat testis (18).

### Sample collection

2 months after injection, the experimental animals were sacrificed under deep anesthesia. The left cauda epididymis and testis were removed. The epididymis tail was used for sperm parameter analysis, and each testis was split into 2 sections. One half was fixed with Bouin's solution for histopathological analysis, and the other half was put in cryotube, then transferred to liquid nitrogen and stored at -80 C for further analysis. For biochemical analyses, blood samples from the aorta were taken.

### Analysis of sperm parameters: motility, count, and viability

The left cauda epididymis was taken out, placed in Ham's/F12 (Gibco, Germany), minced, and incubated at 37 C for 20 min. Next, the sperm sample was placed in a Neubauer's chamber and covered with a cover slide. Then, using the sperm movement pattern, the percentage of sperm motility was assessed (20). To count the sperm cells, the suspension was diluted 1:10 with fixative (1% formalin in PBS). Then a drop of the suspension was transferred to a Neubauer's chamber and examined under a light microscope (21). The viability of the sperm was determined by Eosin-Nigrosin (Merck, Germany) staining. This staining method resulted in the red staining of the dead sperms while leaving the live sperms uncolored (20).

### Histopathology examination

After fixation with Bouin's solution (Merck, Germany), the testis tissue was processed for histologic analysis and then stained with hematoxylin and eosin. In each section (20 microscopic fields), the number of spermatogonia and epithelium thickness were determined at x20 in at least 100 seminiferous tubules for each animal by ImageJ Tools Analysis Software (22). Spermatogonia cells with dark nuclei are found near the basement.

### Hormone analysis

The enzyme-linked immunosorbent assay was used to evaluate the levels of testosterone (DRG, USA) in the plasma according to the manufacturer's instructions (23).

### Quantitative real-time polymerase chain reaction (qRT-PCR)

The expression of *Bax* and *Bcl2* genes, as well as *Cyclo A* (as an internal control) in all experimental groups, were evaluated by quantitative reverse transcriptase polymerase chain reaction (qRT-PCR). For this purpose, total RNA was initially extracted from testis tissue using the RNX-plus reagent (Yekta Tajhiz Azma, Iran) in accordance with the manufacturer's instructions. RevertAid ${\;}^{\text{TM}}$ First Strand cDNA Synthesis kit (Aryatous, Iran) was used to synthesize cDNA from 2 μg of isolated RNA in a 20 μl total volume. The components of the qRT-PCR reaction system were as follow: cDNA, forward and reverse primers solutions at 5 mmol/l, and SYBR green reagent (Yekta Tajhiz Azma, Iran) (Table I). Contains a list of primer sequences. Every sample was run through the LightCyclerⓇ 96 System (Roche, USA) in duplicate. Relative gene expression was then computed CT method (2 ${\;}^{-\Delta \Delta \text{CT}}$ ).

**Table 1:** Primer sequences of used genes

	**Primer sequences (5 ' -3 ' )**		
**Gene**	**Sense**	**Antisense**	**Product length (bp)**
	***Bax***	GCTACAGGGTTTCATCCAG		TCCACATCAGCAATCATCC	174
***Bcl2***	AGCGTCAACAGGGAGATG	CCACAAAGGCATCCCAG	118
***CycloA***	GGCAAATGCTGGACCAAACAC	TTAGAGTTGTCCACAGTCGGAGATG	196
*Bcl2*: B-cell lymphoma 2,* Bax*: *Bcl2*-associated X protein,* CycloA*: Cyclophilin A			

### Ethical considerations

The National Research Council's guidelines were followed during this research. The Arak Ethics Committee, Arak, Iran gave its approval to this research and animal care methods (Code: IR.ARAKMU.REC.1400.053). All procedures were carried out following the ARRIVE guidelines.

### Statistical analysis

GraphPad Prism v9.0.0 (GraphPad Software Inc, CA, USA) was used to conduct all statistical analyses. The results were shown as mean ± standard deviation, interquartile range, and median. The normal distribution of data was evaluated using Kolmogorove-Smirnova. Data were statistically analyzed using a one-way analysis of variance (ANOVA) followed by a Tukey's post-test. P-value < 0.05 was accepted as statistically significant.

## 3. Results

### Morphological characteristics of BM-MSCs

**Figure 1:**
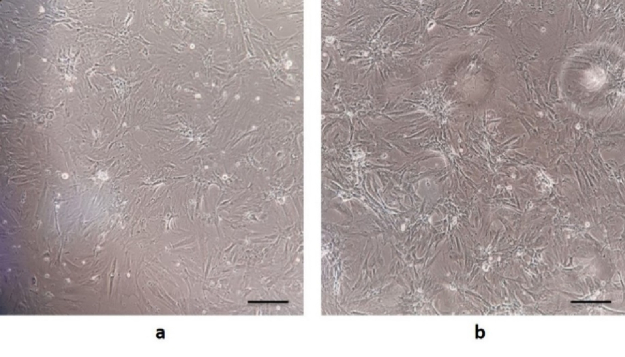
Isolation and culture of bone marrow stem cells. a) BM-MSCs morphology after 24 hr of cell culture, just a few adhering spindle-shaped cells were discovered. b) After 2 wk, some colonies of hundreds of cells were formed. The bar represents 50 μm.

One of the main features of mesenchymal stem cells is their fibroblast-like and spindle-shaped morphology, which was evident in the initial stages of incubation (Figure 1a). The cells initially attached sparsely to the culture flasks, and over time, they began to proliferate and eventually formed tiny colonies. After cultivation for 2 wk, a monolayer and confluence of cells were achieved, and nearby colonies merged throughout the culture (Figure 1b).

### Confirmation of the surface markers

**Figure 2:**
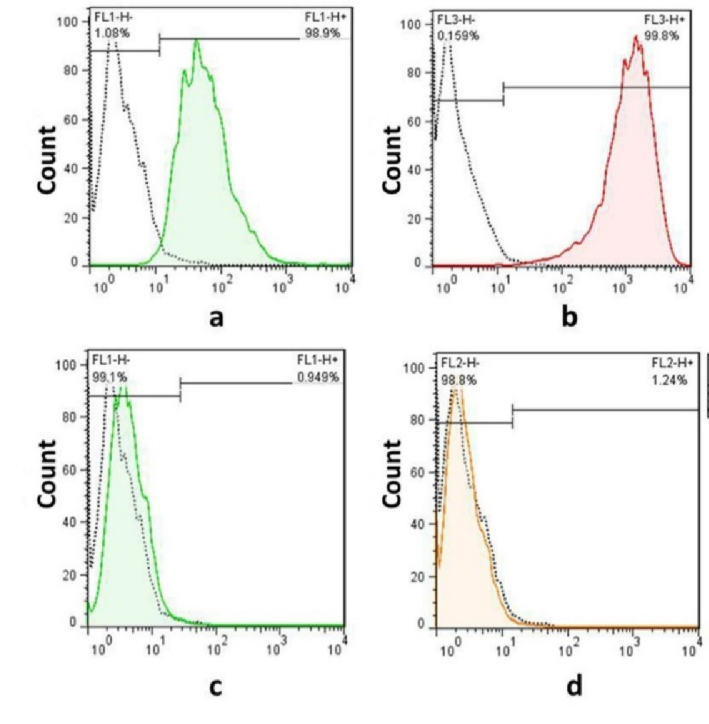
The identification of bone marrow mesenchymal stem cells by flow cytometry. Flow cytometry analysis shows BM-MSCs positive for a) CD44, b) CD90, c) negative for CD34, d) CD45.

In the third passage, the surface markers of BM-MSCs were examined by flow cytometry in order to confirm the isolation of bone marrow stem cells. The acquired cells must express CD44 and CD90 markers but do not express CD34 or CD45 markers (specific markers of hematopoietic cells). Our findings showed that the cultured cells expressed very high levels of CD44 (98.9%) and CD90 (99.1%) (Figure 2a, b). However, the expression of CD34 and CD45 was at a low level, with only 1.24% and 0.94% of the cells expressing these markers, respectively (Figure 2c, d).

### Effects of CM on sperm parameters

Our findings demonstrated that when compared to the control group, all sperm parameters, including concentration, motility, and viability, showed a significant decline (p < 0.001) in both the CP and CP+DMEM groups. However, when condition medium was injected into the efferent tubules, a significant increase was observed in both sperm quantity (p = 0.03) and quality (p < 0.001) in CP-treated rats. No significant difference was observed between sperm parameters in the CP and CP+DMEM groups (Table II).

### Effects of CM on testis histopathology

**Figure 3:**
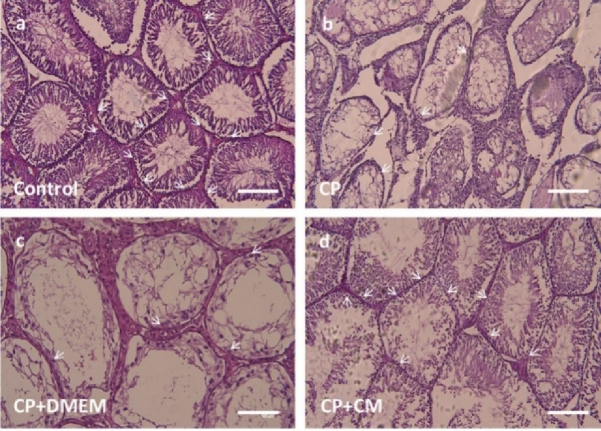
Testis histopathology evaluation by hematoxylin and eosin staining. a) Control, b) CP, c) CP+DMEM, d) CP+CM. Light microscopy of testis tissue shows normal seminiferous tubule orientation containing spermatogonial cells as cells with dark nuclei attached to the basement (arrows) in a and d. In contrast, vacuolization, a decrease in the thickness of seminiferous epithelium and spermatogonial cells (arrows) are found in b and c. CP: Cyclophosphamide, CM: Condition medium, DMEM: Dulbecco's Modified Eagle Medium. Scale bar 50 μm.

**Figure 4:**
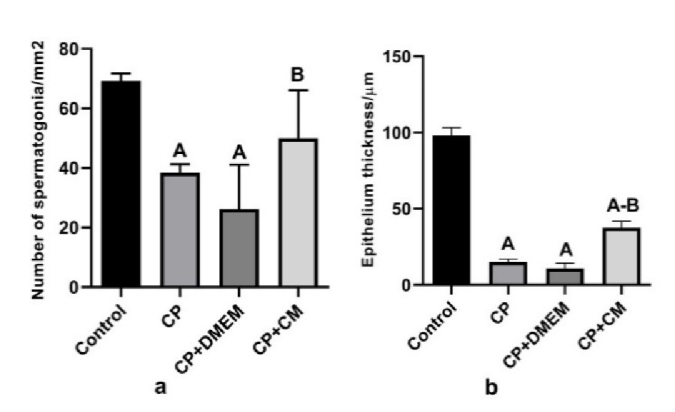
Comparison of the number of a) Spermatogonia and b) Epithelium thickness in different experimental groups. The number of spermatogonia and epithelium thickness increased considerably after CM treatment. A: Significant vs. control, B: Significant vs. CP and CP+DMEM. CP: Cyclophosphamide, CM: Condition medium, DMEM: Dulbecco's Modified Eagle Medium.

According to our histopathological study, there were vacuolization, fewer spermatogonia (p < 0.001) and a thin epithelium (p < 0.001) in all of the rats given CP or CP+DMEM. The animals that received CM showed testicular morphologic features similar to those in the control group. The seminiferous tubules showed normal orientation containing spermatogonial cells as cells with dark nuclei attached to the basement in the control and CP+CM groups (Figure 3). These animals exhibited a significant increase in spermatogonia count (p < 0.001) (Figure 4a) and seminiferous epithelium thickness (p = 0.02) (Figure 4b) in comparison to the CP and CP+DMEM groups.

### Effects of CM on testosterone levels

**Figure 5:**
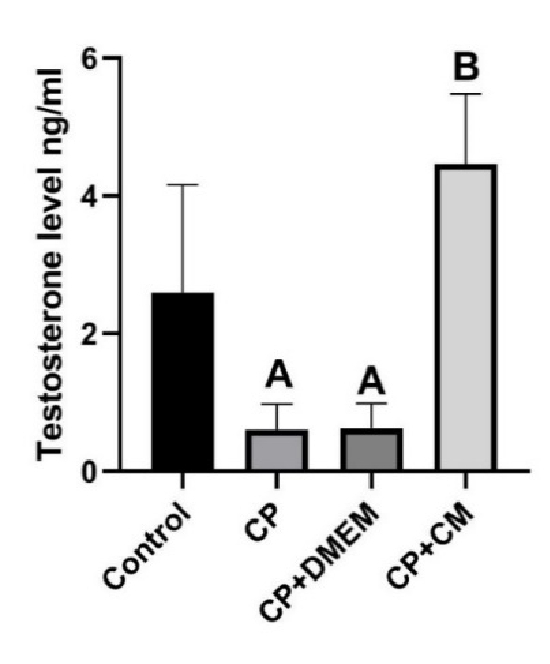
Level of testosterone hormone 2 months after BM-MSC-CM injection. The CM treatment group had significantly higher serum testosterone levels than the CP and CP+DMEM groups. A: Significant vs. control, B: Significant vs. CP and CP+DMEM. CP: Cyclophosphamide, CM: Condition medium, DMEM: Dulbecco's Modified Eagle Medium.

Our results revealed that rats treated with CP and CP+DMEM had considerably lower plasma testosterone levels compared to the control group (p = 0.02). However, injection of CM into CP-treated rats resulted in a notably higher level of testosterone in their plasma (p < 0.001) (Figure 5).

### Effects of CM on gene expression

**Figure 6:**
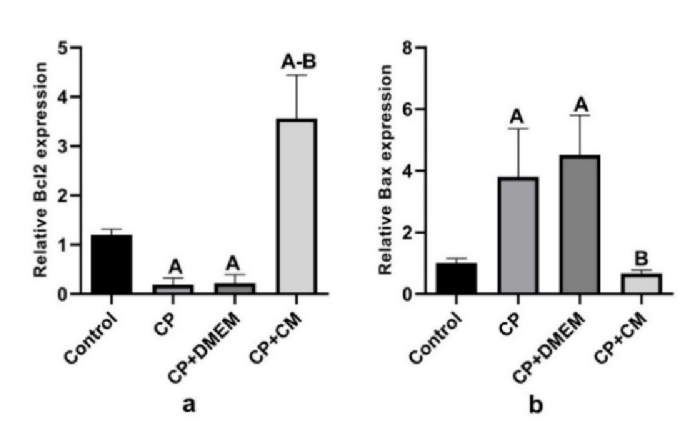
Real-time PCR analysis of a) *Bcl2* and b) *Bax* mRNA expression in different experimental groups. Following treatment with CM, the expression of *Bax* drastically reduced while considerably increased the expression of *Bcl2*. A: Significant vs. control, B: Significant vs. CP and CP+DMEM. CP: Cyclophosphamide, CM: Condition medium, DMEM: Dulbecco's Modified Eagle Medium.

We investigated whether the injection of CM into the efferent tubules of CP-treated animals could reduce apoptosis in the testis tissue. For this purpose, the gene expression of *Bcl2* (antiapoptotic) and *Bax* (proapoptotic) was examined. Our findings revealed that the expression of *Bax* was considerably increased in the CP (p < 0.001) and CP+DMEM (p < 0.001) groups compared to the control group, while the mRNA level of *Bcl2* was downregulated (p = 0.01). Injection of CM could dramatically upregulate the antiapoptotic (p < 0.001) and reduce the proapoptotic (p < 0.001) genes compared to the CP and CP+DMEM groups. Notably, *Bcl2* was expressed more in the CP+CM group than in the control group (Figure 6).

**Table 2:** Sperm parameter analysis in different experimental groups

**Variables**	**Control**	**CP**	**CP+DMEM**	**CP+CM**	**P-value**
**Count × 10^6^**	43.6 ± 6.5 (42, 44)	26.25 ± 7.5 (25, 26.87) ${\;}^{\text{a}}$	27 ± 8.53 (27.5, 25.62) ${\;}^{\text{a}}$	37.5 ± 4.7 (38.5, 37) ${\;}^{\text{b}}$	< 0.001* < 0.001** 0.03*** 0.03****
**Viability%**	81.58 ± 5.5 (82.35, 81.39)	31.76 ± 12.58 (29, 31.13) ${\;}^{\text{a}}$	34.43 ± 11.93 (29.45, 39.92) ${\;}^{\text{a}}$	70.56 ± 5.58 (69.28, 71.20) ${\;}^{\text{b}}$	< 0.001* < 0.001** 0.003*** 0.003****
**Progressive%**	61.0 ± 7.41 (60, 61)	16.25 ± 6.29 (15, 16.8) ${\;}^{\text{a}}$	14.25 ± 4.34 (13.5, 14.62) ${\;}^{\text{a}}$	59.50 ± 4.2 (59, 59.75) ${\;}^{\text{b}}$	< 0.001* < 0.001** < 0.001*** < 0.001****
**Immobility %**	7 ± 2.73 (5, 7.5)	93.33 ± 11.55 (100, 90) ${\;}^{\text{a}}$	85.13 ± 17.61 (87.5, 83.94) ${\;}^{\text{a}}$	17.50 ± 6.45 (17.5, 17.5) ${\;}^{\text{b}}$	< 0.001* < 0.001** < 0.001*** < 0.001****
All data were shown as Mean ± SD (MD, IQR). One-way ANOVA followed by a Tukey's post-test. a) Significant vs. control, b) Significant vs. CP and CP+DMEM. *: Control vs CP, **: Control vs CP+DMEM, ***: CP+CM vs CP, ****: CP vs CP+DMEM. CP: Cyclophosphamide, CM: Condition medium, DMEM: Dulbecco's Modified Eagle Medium					

## 4. Discussion

This research has shown that injecting BM-MSC-derived condition medium into the efferent duct of CP-treated rats could improve sperm parameters, enhance testicular histopathology, and reduce apoptosis. Following chemotherapy, the testicles may experience structural and functional issues, such as a decrease in sexual hormones and a reduction in the number of germ cells, Sertoli cells, and Leydig cells, ultimately leading to infertility (1).

It is widely known that both radiation and chemotherapy can have negative impacts on male fertility during the pre-pubertal and pubertal years. While it is currently possible to preserve the fertility of adult patients by freezing their sperm before undergoing chemotherapy or radiation treatment, techniques for preserving the fertility of pre-pubescent cancer patients who have not yet started producing sperm are still in the experimental stage (24, 25). CP is an alkylating medication that targets proliferating cells and prevents DNA synthesis, leading to negative impacts on reproduction (26). The activation of p53 is a critical step in the induction of apoptosis by CP. DNA damage leads to the activation and stabilization of p53, which acts as a transcription factor. Activated p53 induces the expression of several proapoptotic genes, such as *Bax*, while repressing the expression of antiapoptotic genes, such as *Bcl2*. This transcriptional regulation shifts the balance toward apoptosis (27).

The findings demonstrated that the use of CP can have detrimental effects on testosterone levels and sperm quality, resulting in a decrease in the number of spermatogonia and epithelial thickness in seminiferous tubules. Moreover, *Bax* expression increased, while *Bcl2* mRNA level dropped after CP administration. Previous studies have indicated that CP alters the structure of the basic proteins in sperm heads, increases the rate of malformed sperm, and causes biochemical and histological changes in the testis (28, 29). Patients treated with CP exhibited a marked decrease in testicular weight and histological abnormalities, along with a reduction in germ cells, as indicated by testicular weight loss. The decrease in gonadotropin and testosterone levels, which influence spermatogenesis, may contribute to this reduction (30, 31). CP can damage Leydig cells by releasing free radicals, leading to a significant drop in testosterone levels as a negative side effect. It has been proven that Sertoli cells play a crucial role in spermatogenesis, as well as in the structural growth and maturation of germ cells. The disruption of the connection between testosterone-dependent Sertoli cells and germ cells may result in their disintegration, potentially contributing to the failure of spermatogenesis in males treated with CP (31). Furthermore, previous research has shown that CP promotes apoptosis at specific times during the germinal cycle. Consequently, cell death in spermatogenic cells could be a contributing factor to the decrease in epididymal sperm count (32).

In this study, our aim was to reduce the side effects of CP on testicular tissue by injecting CM derived from BM-MSCs into the efferent duct. To achieve this, we isolated and cultured BM-MSCs, and confirmed their capacity by demonstrating their ability to express high levels of CD44 and CD90 (19). After collecting the CM, we injected it into the efferent duct of CP-treated rats. 2 months after the CM treatment, the rats showed improved sperm parameters, including motility, number, and viability, as well as improved testicular histopathology and an increased level of testosterone. Additionally, there was an increase in the expression of genes that inhibit apoptosis. The importance of the secretome in tissue regeneration has recently garnered more attention (7, 33). Previous studies have shown that growth factors released by BM-MSC can alleviate testicular damage resulting from chemotherapy (4, 5).

This approach is entirely reliant on the soluble substances produced by the cells. It is based on the generation and release of nutritional factors or chemokines (34). After a few days of cultivation, each cell population secretes growth factors into the surrounding medium. In this technique, the soluble components of CM can initiate regenerative signaling pathways without the assistance of cells (7). Previous studies have demonstrated the effectiveness of CM derived from mesenchymal stem cells in alleviating apoptosis and histological abnormalities in ischemic/reperfusion animal models (35) and busulfan-treated mice (10). The protective effects of CM on busulfan-induced testicular damage were investigated and immediately after busulfan injection, CM was injected directly into the testis, which is different from our study where we administered CM through the efferent ducts 2 wk after CP injection (10). Sharifian et al. injected CM in animals undergoing ischemia/reperfusion which involves a different pathogenesis (35). BM-MSCs are considered to be a promising source for treating testicular cells, since they produce the necessary growth factors required for restoring fertility (5).

As a result, using BM-MSCs-derived CM may be a successful method for restoring spermatogenesis after chemotherapy. Saleem et al. analyzed the CM produced by human BM-MSCs and found that it contains some components that can reduce oxidation, inhibit apoptosis, and control cell growth. According to the proteomics analysis, this CM contains several antioxidant enzymes, such as glutaredoxins, peroxiredoxins, and superoxide dismutase, which are also important in decreasing apoptosis (36). Additionally, BM-MSCs secret important growth factors like vascular endothelial growth factor, angiopoietin-1, basic fibroblast growth factor, and hepatocyte growth factor that have been proven to have remarkable contributions on reparative and regenerative processes in damaged tissues. These growth factors can activate signaling pathways, such as the PI3K/AKT and MAPK/ERK pathways, which promote cell survival and inhibit apoptosis (5).

This study highlights the beneficial effects on new vascularization, stimulation of cell differentiation, and proliferation (37). Our findings from sperm parameters, hormonal examination, as well as histology and gene expression assessment demonstrate the effectiveness of these cytokines on testis tissue following the injection of CM into rats treated with CP. This confirms the positive impact of the components of the CM on regenerating damaged tissue. Nevertheless, several limitations of this study should be noted. Firstly, it is important to consider that the results obtained using an animal model may not be directly applicable to humans, and further studies are needed to determine the extent of this applicability. Secondly, we measured the changes in mRNA expression levels, but we did not conduct additional techniques such as immunohistochemistry or Tunnel assay to determine changes in protein levels and cell death.

## 5. Conclusion

In conclusion, our data explains that CM derived from BM-MSCs possesses significant therapeutic potential in ameliorating testicular damage induced by CP. It is anticipated that stem cell CM could serve as a promising alternative to stem cell transplantation, thus addressing the limitations associated with this approach. Ultimately, our results provide some evidence for the development of a novel treatment for testicular damage based on stem cell CM.

##  Data availability

The data that support the findings of this study are available from the corresponding author upon reasonable request.

##  Author contributions

All authors contributed to the study conception and design. Z. Esmaeelpour, S. Madadi, and M. Baazm performed material preparation, data collection, and analysis. The first draft was written by Z. Esmaeelpour, and all authors commented on the previous version of the manuscript. All authors read and approved the final manuscript.

##  Acknowledgments

The current study emanated from a proposal approved and supported by the Arak University of Medical Sciences, Arak, Iran. It was granted by the council of Arak University of Medical Sciences, Arak, Iran (grant number: 6422).

##  Conflict of Interest

The authors declare that there is no conflict of interest.
